# A process evaluation: Does recruitment for an exercise program through ethnically specific channels and key figures contribute to its reach and receptivity in ethnic minority mothers?

**DOI:** 10.1186/1471-2458-13-768

**Published:** 2013-08-19

**Authors:** Marieke A Hartman, Vera Nierkens, Stephan W Cremer, Karien Stronks, Arnoud P Verhoeff

**Affiliations:** 1 Department of Public Health, Academic Medical Center, University of Amsterdam, Meibergdreef 9, AZ 1105 Amsterdam, the Netherlands; 2 Department of Epidemiology, Documentation, and Health Promotion, Public Health Service of Amsterdam, Nieuwe Achtergracht 100, WT 1018 Amsterdam, the Netherlands; 3 Department of Behavioral Sciences and Health Promotion, University of Texas School of Public Health, 7000 Fannin, Houston, TX 77054, USA; 4 Department of Sociology and Anthropology, University of Amsterdam, Oudezijdse Achterburgwal 185, DK 1012 Amsterdam, the Netherlands

**Keywords:** Recruitment, Recruiter, Channel segmentation, Community health worker, Ethnic minorities/migrants, Health promotion, Cultural targeting, Exercise/physical activity

## Abstract

**Background:**

Ethnic minority women from low-income countries who live in high-income countries are more physically inactive than ethnic majority women in those countries. At the same time, they can be harder to reach with health promotion programs. Targeting recruitment channels and execution to ethnic groups could increase reach and receptivity to program participation. We explored using ethnically specific channels and key figures to reach Ghanaian, Antillean, and Surinamese mothers with an invitation for an exercise program, and subsequently, to determine the mothers’ receptivity and participation.

**Methods:**

We conducted a mixed methods process evaluation in Amsterdam, the Netherlands. To recruit mothers, we employed ethnically specific community organizations and ethnically matched key figures as recruiters over Dutch health educators. Reach and participation were measured using reply cards and the attendance records from the exercise programs. Observations were made of the recruitment process. We interviewed 14 key figures and 32 mothers to respond to the recruitment channel and recruiter used. Content analysis was used to analyze qualitative data.

**Results:**

Recruitment through ethnically specific community channels was successful among Ghanaian mothers, but less so among Antillean and Surinamese mothers. The more close-knit an ethnic community was, retaining their own culture and having poorer comprehension of the Dutch language, the more likely we were to reach mothers through ethnically specific organizations. Furthermore, we found that using ethnically matched recruiters resulted in higher receptivity to the program and, among the Ghanaian mothers in particular, in greater participation. This was because the ethnically matched recruiter was a familiar, trusted person, a translator, and a motivator who was enthusiastic, encouraging, and able to adapt her message (targeting/tailoring). Using a health expert was preferred in order to increase the credibility and professionalism of the recruitment.

**Conclusions:**

Recruitment for an exercise program through ethnically specific organizations seems to contribute to its reach, particularly in close-knit, highly organized ethnic communities with limited fluency in the local language. Using ethnically matched recruiters as motivator, translator, and trusted person seems to enhance receptivity of a health promotion program. An expert is likely to be needed for effective information delivery.

## Background

In high-income, Western countries, non-Western ethnic minority groups have been found, on average, to be less physically active than the host population [1,2]. Specifically, they tend to exercise less during leisure time [3,4]. As the Western countries become increasingly more ethnically diverse [5], there is a need to promote physical activity within multiethnic populations. However, it can be a challenge for health professionals to reach ethnic minority groups with health promotion programs, such as exercise programs [6,7].

To increase this reach, and thereby the accessibility of health promotion in the target group, employing an ethnic group’s community resources is a promising recruitment method. Examples of such resources are churches, local community leaders and organizations, ethnically specific media, networks, and events [7,8]. A health worker from the community would be trusted (i.e., source similarity) [9] and could, therefore, be key to recruiting community members. In contrast, health providers may be seen as outsiders and less trustworthy [10].

However, not all prior studies support using ethnically specific community resources for recruitment. Formative studies among different ethnic groups have shown that some ethnic minority groups seem to rely less on ethnically specific resources than others [11,12]. Moreover, a systematic review that compared four different recruitment methods for reaching ethnic minorities – social marketing, referrals, healthcare, and community outreach – found community outreach to be the least successful in terms of percentages [7].

Therefore, the utility of ethnic group’s community resources for improving participation in health promotion programs is not yet clear and merits further study [7]. Two questions remain: first, do ethnically specific community resources contribute to recruitment and second, if so, under which conditions (why, how)? As to community recruitment resources, one can distinguish channels and recruiters [13]. The former is thought to affect reach – in other words, contact with the target population [13]. The latter, along with the messages applied, has been suggested to affect receptivity and the decision to participate [13,14].

This study aims to test the potential utility of targeting recruitment channels and recruiters to specific ethnic minority groups, with a particular focus on the conditions and mechanisms underlying their impact. We focused on mothers of young children because this group is at increased risk of physical inactivity [2,15]. The main research questions were 1) could ethnically specific channels successfully contribute to the reach among mothers from three ethnic minority groups and if so, why, and 2) do ethnically matched recruiters (key figures) contribute to these mothers’ receptivity and subsequent participation in an exercise program compared with recruitment by ethnic Dutch health educators and if so, how?

## Methods

We conducted a mixed methods process evaluation of an ethnicity-based targeted recruitment on reach, receptivity, and final participation in the exercise program “Big Move *mama*”. Big Move *mama* was an existing program that suited the shared needs of mothers from a multiethnic population. These shared needs were derived from formative research [16] (Hartman, Nierkens, Nicolau, Hosper, Cremer, Stronks: Grounding weight-related health promotion for a multiethnic population of mothers: an analysis of similarities and differences in perceived determinants, in preparation; Hartman, Verhoeff, Cremer, de Schouwer, Stronks, Nierkens: Evaluating ‘Big Move *mama*’: a general exercise program for a multiethnic population of mothers, in preparation). We defined “reach” as the extent to which the health educator came into contact with the target group. “Receptivity” was defined as the extent to which the target population understood, was open to and paid attention to the information, expressed a positive attitude, and seemed interested. We considered a mother to be a participant if she passed the intake interview with the Big Move *mama* coach and enrolled in more than one exercise class.

**Figure 1:**
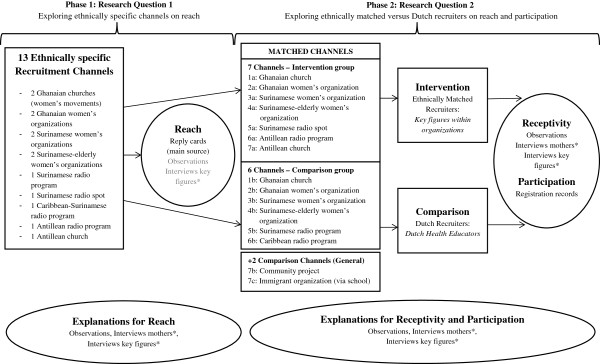
**Research design recruitment for Big Move *mama: *Input, output, and data collection tools.**

In phase 1 of this study, ethnically specific channels, like community organizations, were used to reach mothers from three ethnic minority groups. Then in phase 2, two conditions were compared regarding the recruiter. For this purpose the recruitment channels were matched according to organizational characteristics, such as type, ethnic target group, how organized (e.g., weekly meetings versus ad-hoc activities), and how information could be communicated (e.g., group education versus radio interview). In half of the organizations, mothers were recruited by an ethnically matched recruiter, a key figure within the collaborating community organizations (intervention condition). In the other half, mothers were recruited by ethnic Dutch health educators (comparison condition). This study design (Figure 1) was submitted to the Medical Ethical Review Committee (METC), which confirmed that the study required no further assessment or approval by that Committee because the Dutch Medical Research Involving Human Subjects Act does not apply.

### Study context: location and target group

**Figure 2:**
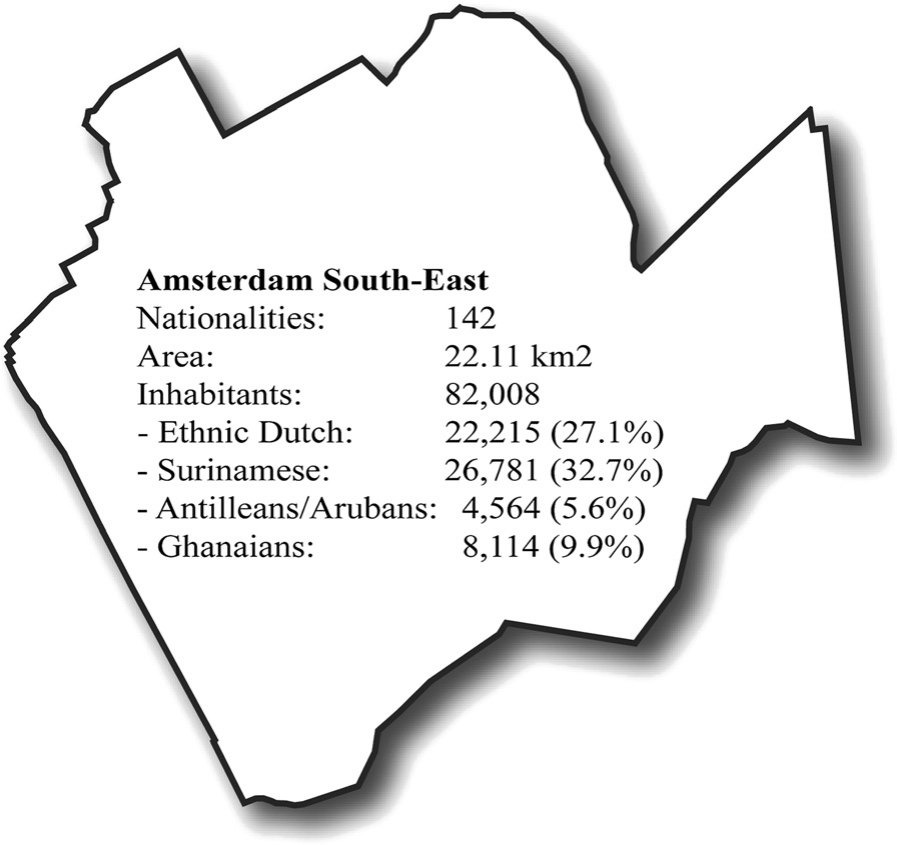
**Sociodemographic characteristics study location Amsterdam South-East.** Key figures 2011 derived from the Department of Research and Statistics, Municipality of Amsterdam [22].

The process evaluation was conducted in Amsterdam South-East, an ethnically diverse district in which Ghanaians, Afro-/Hindustani Surinamese (hereafter referred to as Surinamese), and Antilleans/Arubans (hereafter referred to as Antilleans) are the largest ethnic groups (see Figure 2). Residents of Amsterdam South-East have the highest obesity and physical inactivity levels in Amsterdam [17,18]. In spite of this, possible ways to involve the main ethnic groups living in this district for health promotion programs have been understudied compared with other ethnic minority groups living in the Netherlands, such as Moroccan, Turkish, and Cape-Verdean [19-21].

Ghanaians, Surinamese and Antilleans living in the Netherlands differ in socioeconomic status, language, and migration history. The Ghanaians are known as hard working but have relatively marginalized positions, while Surinamese women have a favorable position on the labor market. Although English is the official language in Ghana, there are 75 Ghanaian languages and dialects. Ghanaians tend to have poor Dutch language proficiency. Antillean and Surinamese women have less difficulty learning Dutch. Though, Dutch language proficiency is best among Surinamese; Antilleans use their own language more [23]. The Netherlands Antilles and Suriname have a colonial history with the Netherlands. Moreover, most Surinamese emigrated before 1976, so many now living in the Netherlands are second generation [24].

### Ethnically specific recruitment channels

Initially we mapped the existing, ethnically specific organizations using the Internet, experiences of the Public Health Service that collaborate with the organizations, and snowball methodology. Our preference was to collaborate with women’s organizations/groups that met regularly or ad-hoc; if these were not available or resulted in a low reach, ethnic-audience oriented radio was added as a channel (Figure 1, phase 1).

Recruitment through ethnically specific channels was perceived as successful reach if the target number of 27 mothers per ethnic group was reached within 4 months (November 2010–February 2011). The target number was based on the desired number of participants and an estimate of those who would refuse participation. To fill the two available Big Move *mama* groups, we aimed to recruit 30–40 participants, equally distributed among the three ethnic groups. To achieve this, we expected we would need to reach 80–100 mothers to account for those uninterested (50%), and those interested but who ultimately would not enroll (25%). Based on earlier collaborations with community organizations by the Public Health Service of Amsterdam, we expected that four organizations per ethnic group would be sufficient to attain this goal.

### Recruiters and recruitment method applied: ethnically matched key figures versus Dutch health educators

To evaluate the process, in phase 2, the collaborating organizations were matched to study ethnically specific key figures versus Dutch health educators as recruiters. Further, two additional general audience-oriented channels that used the same kind of recruitment approach were employed, an immigrant organization that recruited via a school and a community project. We were able to match all other community organizations according to type, ethnic group served, how organized, and type of information given, with two exceptions. A Surinamese radio spot was compared with a radio interview of a health educator, and an interview on a Surinamese program on Caribbean radio was compared with one on an Antillean radio program (Figure 1, phase 2).

The recruiters for half of the channels were ethnically matched key figures from the collaborating community organizations. They acted as community health workers to inform the women about the benefits of exercise and to invite them to take part in Big Move *mama*. Although some figures had collaborated on Public Health Service projects (e.g., on HIV/STI prevention), they did not have official roles as community health workers; above all they were the leaders within their organizations. If organization leaders were not confident about recruiting, either another key figure within the organization was assigned, or a health educator supported them in providing the most important information. Recruiters for the other half of the channels were two female ethnic Dutch health educators from the Public Health Service of Amsterdam.

The general basis of the recruitment method consisted of three contact moments per organization and was adapted to the organizational characteristics of the pairs. Hence, intensity could differ between pairs but not within pairs. First, to increase attention, the information session about Big Move *mama* was announced during a prior meeting or via pamphlets/mailing. Second, the actual information giving and recruitment took place at group meetings, one-to-one or via radio interviews/a spot. A brochure with a reply card and a pen were manually distributed to the mothers of young children and collected at the end of the meeting. Mothers could indicate on the reply card whether they would not, might, or wanted to join Big Move *mama*. Mothers who heard a radio spot could call a key figure and/or the health educator. Third, a Dutch health educator made a phone call to confirm the mother’s interest in participating, for final decision-making, and to schedule an intake interview for participants with the Big Move *mama* coach. This proactive approach by the health educator was meant to increase accessibility.

The actual information giving and recruitment was preferably done face-to-face, with an interactive component to promote information processing and to create positive modeling within the group (e.g., by asking who already had exercise experience and what the advantages were). The core recruitment message included mother-specific motivators and enablers that were shared by the different ethnic groups [25-27] (Hartman, Nierkens, Nicolau, Hosper, Cremer, Stronks: Grounding weight-related health promotion for a multiethnic population of mothers: an analysis of similarities and differences in perceived determinants, in preparation). An example: “We have an exercise program especially for mothers – although mothers always tend to be busy taking caring of others, it’s also important to take care of yourself. Enjoy some time for yourself – you deserve it!” Moreover, we emphasized that childcare would be provided.

### Data collection

Recruitment evaluation was based on both quantitative and qualitative data collection. To evaluate ethnically specific channels on reach, a quantitative impression of reach was generated using reply cards and logging of phone calls received by the health educator. Qualitatively, a health education and a key figure who had been present estimated the number of mothers present during the information giving session. Explanations for reach were derived from observations, interviews with mothers, and interviews with key figures (Figure 1). Table 1 presents the observation scheme that was used and two topic lists that guided the interviews. These included topics about alternative channels as a possible explanation for the found reach via ethnically specific channels.

**Table 1:** Observation scheme and topic lists for key figures and target population

**Topic**	**Observation scheme**	**Topic list: key figures interviews**	**Topic list: target population (mothers) interviews**
Reach	*During recruitment*:	What is your estimate of the number reached, and the number of mothers with young children in this group?	
	Number of attendees?		
	Proportion of mothers with young children from South-East/others?		
	Gender distribution?		
	Proportion overweight/not overweight?		
	Ethnicity?		
	Estimated educational level?		
> Explanation for reach	*Before recruitment*:	Did you expect this reach/number of attendees?	How did you first hear about Big Move *mama*? (which organization/radio station/person? )
	What kind of organization?	- Yes/higher/lower	- How did you feel about hearing more about Big Move *mama* through …?
	Size of the organization?	- Why do you think so many mothers were reached?	- What is your relationship with that organization/person?
	Fixed group?	◦ Own role and approach	◦ Aim of going to this organization?
	Fixed meeting(s)?	◦ Organizational approach	◦ Activities?
	Target group: women/mothers?	◦ Information/topic	
	Target group: South-East?	◦ Strategy used	
	Health focus?	◦ Mothers’ general motivation to attend/reactions	
	Additional characteristics?	◦ Contextual factors (e.g., organizational structure of community)	
> Alternatives to increase reach	Side effects/tips for other channels?	How could the reach among [Surinamese/Ghanaians/Antilleans] have been improved?	How do you normally hear about news and activities in the neighborhood? How do you find out?
		Do you have tips about other channels?	
Receptivity	What is the effect of the key figure role? How does the group respond to this?	What do you remember about the information given?	What did you think about Big move *mama* when you heard more about it?
	How does the group react to the Dutch health educator?	What do you remember about the recruiter?	- Did you immediately decide whether you wanted to join Big Move *mama*? Or did you think about it first?
	*(e.g., understanding, open, attention, appreciation, acceptance)*		
	Which questions are asked?		
	Which responses are given?		
> Explanation for receptivity and participation	Which role does the key figure play?	Did you expect so many mothers to sign up for Big Move *mama*?	Who told you more about Big Move *mama*? (PHS health educator/intermediary from the organization)
	What degree of respect does the key figure command?	- Yes/more/less	- Can you tell me more about the woman who gave the information?
	How does the message come across?	- Why did you think so many mothers signed up?	- What did you think about the person who gave the information? (ask follow-up questions only if answered with good, nice, etc.)
	- About exercising	◦ Own role and approach	- Who do you think is the best person to tell more about Big Move *mama*?
	- About Big Move *mama*	◦ Role and approach of Dutch health educator	◦ From which organization?
	How is the message communicated? (e.g., only by providing information, with an interactive approach, etcetera)	◦ Information	◦ Why?
	Context factors?	◦ The program Big Move *mama*	What did you think about the information you received about Big Move *mama*?
	- Duration of information giving	◦ Strategies used	How do you prefer to receive information?
		◦ Mothers’ expectations, reactions, and own motivations/barriers	- In which language?
		◦ Contextual factors	- In which form: spoken, written, as visuals, etc.
		What motivated you to collaborate and invest in spreading the word about Big Move *mama*?	What was your reason or reasons to participate or not?
		Were there also factors that demotivated you?	
		Did you feel you were able to meet the expectations of the Public Health Service?	
		- Why did/didn’t you?	

To evaluate using ethnically matched recruiters versus Dutch health educators on receptivity and participation, participation was measured quantitatively using enrollment and attendance records of the Big Move *mama* program. The qualitative data collection tools (observations, interviews with mothers and key figures) were used to gather insight into receptivity first. Indicators of receptivity included an open, positive atmosphere – derived from body language – and active, positive responses to the recruiter during interaction or by asking questions out of interest. Second, these data collection tools explored explanations for receptivity and participation (Figure 1, Table 1).

The observation schemes were completed by one (MH) or both Dutch health educators during or directly after the recruitment activities. MH conducted the semi-structured interviews with the key figures, regardless of whether they were a contact person (N = 7) or a recruiter (N = 7) Further exploratory questions for the key figures, in addition to the standard topic list, were based on the health educators’ observations and previous interviews. Semi-structured interviews were conducted with a selection of the reached mothers by independent interviewers who had not been involved in recruitment. In total, 32 mothers were interviewed, 21 who were recruited via the intervention condition by a key figure, and 11 who were recruited via the comparison condition by a health educator. Five of the 32 mothers, all Surinamese, were included via general audience-oriented channels that were used to enable phase 2. Twenty of the 32 had enrolled in the exercise program (i.e., participants), 12 were non-participants. The interviewees represented a diverse population; they differed, for example, on education level, Dutch language proficiency, and time since migration (Table 2).

**Table 2:** Characteristics of interviewed key figures and mothers

	**Ghanaian**	**Antillean**	**Surinamese**
**No. of key figures**	***n*** **= 4**	***n*** **= 4**	***n*** **= 6**
**Represented organizations**			
Ethnically specific organizations/media	2 churches 2 women’s organizations	1 church 1 organization that used radio	4 women’s organizations: 3 also used radio, 1 also via church and schools
General audience-oriented channels		1 immigrant organization*	1 community project*
Intervention group	2	2	2
Control group	2	1	3
			
**No. of mothers**	*n* = 20	*n* = 4	*n* = 8
Intervention group	14	4	3
Comparison group	6	-	5
Participants	13	3	4
Non-participants	7	1	4
Mean age	42 (25-50)	43 (33-53)	36 (25-50)
*Migration background*			
Generation: 1st	20	4	4
Mean age of migration (range)	24 (16-36)	26 (10-47)	12 (4-23)
Mean time since migration (range)	19 (11-25)	18 (6-23)	24 (17-33)
Missing age and time since migration	-	-	1
*Dutch language proficiency*			
Good/native	7	4	8
Poor/average	13	-	-
*Ethnic identity*			
More Dutch	2	1	1
50/50	15	1	7
More Ghanaian/ Antillean/ Surinamese	3	2	-
*Religion*			
Regular church attendance (once/week)	17	3	3
Less-frequent church attendance/ not religious	1	1	3
Missing	2	-	2
*Education*			
Primary school	5	-	-
Junior secondary school	5	2	-
Senior secondary school	5	-	-
Senior secondary vocational education	5	2	6
Higher professional education /university	-	-	2
*Employment*			
Yes	14	2	7
No	6	2	1

* These were the additional general audience-oriented organizations employed to reach more mothers to explain differences in reach and to enable studying research question 2, they are arranged in the Table by ethnicity of the organization leader (key figure).

In advance of the interviews, interviewees consented to the taping of the interviews and participation in the study. Anonymity of the transcripts and reporting was assured. The interviews with mothers lasted 30–35 minutes and 75 minutes for key figures. The mothers received a €10 gift voucher for participating. Key figures received a financial incentive for their organizations.

### Data analyses

The quantitative data on reach and participation were analyzed using descriptive analyses. The qualitative data on receptivity, as well as on the explanations for reach, receptivity, and participation were analyzed using content analyses. To increase validity, two coders – a health educator (MH) and an independent coder not involved in the study (CV) – coded the first interviews with mothers and key figures independently, compared their results, and discussed the differences. This was an iterative process until inter-coder consensus was reached (i.e., the same excerpts were extracted and assigned to the same key codes for reach, receptivity, or participation and to similar sub-codes for explanation types). Then, the health educator coded the final interviews.

All data resources were charted per channel (columns) and study outcome (rows; i.e., reach, explanations for reach, alternative channels, receptivity, explanations for receptivity, participation, and explanations for participation). This enabled us to analyze the data from different resources on contradiction, confirmation, and complementation/explanation. Moreover, data could be analyzed between channels and, so, ethnic groups, as well as patterns across channels/ethnic groups to explain reach, receptivity, and participation. In addition, with regard to research question 2, data were compared between the intervention and comparison condition per pair, then patterns were analyzed across pairs to see which dominant explanatory themes arose. We adjusted for pairs that were not perfectly matched by only using outcomes that confirmed or complemented results of the well-matched pairs.

## Results

### Reach: contribution of ethnically specific channels

Collaboration with 13 ethnically specific organizations reached 50 mothers in total. The success of using ethnically specific channels varied between the targeted ethnic communities: 37 Ghanaians, 6 Antilleans, and 6 Surinamese mothers were reached (Table 3).

**Table 3:** Reach to mothers per ethnically specific channel according to ethnicity

**Ethnically specific channel**	**Ghanaian**	**Antillean**	**Surinamese**
Churches	13	5	n.a.
Women’s organizations	24	n.a.	5
Radio	n.a.	1	1
***Total reach***	37	6	6

*n.a.*, not applied or applicable.

The differences between ethnic groups seem to be related to the organizational structure within the communities. If there were more organizations available, and if these had fixed groups that met at fixed times and places and also included young adult women (≈25–50 years of age), then more mothers with young children were reached with information about Big Move *mama*. We mapped many Ghanaian organizations, especially churches (*n* > 5), that met our criteria. Collaboration with two Ghanaian churches and two women’s organizations resulted in the 37 women reached. There were also several Surinamese organizations, but only two held regular meetings which were aimed at women 50 years and older. We collaborated with 4 Surinamese women’s organizations, of which a key figure also approached two Surinamese schools and a church, and three were involved into the employment of Surinamese radio programs/stations. This resulted in the 6 mothers reached. There were only four Antillean organizations identified, of which two collaborated. Only a church had regular meetings with fixed groups, which accounted for 5 of the 6 Antillean women reached.

Our explanation for these differences was supported and further explained by the interviews with mothers and key figures. Ghanaian key figures frexplained that the many regular gatherings of Ghanaian women, which resulted in the successful reach, stemmed from their community’s close-knit nature and retained culture and that they find the Dutch language more difficult to learn.

I: “The Ghanaian women get together quite often, in fixed groups. Why do you think this is? In your group, in churches…”

R: “In churches, yes… we love getting together, and our own things”. (Ghanaian key figure)

R: “It’s very nice, getting the women together and then giving them some information about… Dutch society. A lot of women have been here for 25, 30 years, but because they can’t read or write, they miss out on certain things”. (Ghanaian key figure)

Accordingly, the answers of the Ghanaian mothers indicated that they continue to use their own channels. They mentioned almost exclusively Ghanaian-specific channels for obtaining information about neighborhood activities (e.g., other churches, another organization, television, radio, and word of mouth within the community). Information obtained through these organizations was described as being better received than that obtained from mailings and easier to access than information obtained through a general channel (e.g., family physician). Information received through Ghanaian-specific channels was clearer because it is communicated in their own or second language (i.e., English) and their questions could be answered. Women mentioned that this clarity resulted in trust and confidence and made it easier to enroll in activities (“*Then you know what to do*”).

I: “Was it good that someone came [to your organization] to talk about [Big Move *mama*]?”

R: “Yes, then you can hear it, because sometimes… although you might want to read [it], you can’t… or you *are* able to read [it] but you don’t understand it very well. (…) It’s very important to me. It gives you [a] good feeling, you can trust it”. (Ghanaian mother)

In contrast, for the Antillean and Surinamese women, the Dutch language posed no problem. Furthermore, key figures described the Surinamese and Antillean women as not wanting obligations and not being very close with each other (only with their families). The latter was directly linked by these key figures to fewer regular community gatherings thus reach was seen as more difficult.

I: “We saw how the Ghanaian women got together a lot, they really got together in groups that met every two weeks. Isn’t there somewhere like that where we could reach the Antillean group all at once?”

R: “No, Antillean mothers are very different than Ghanaian mothers. You can’t compare them”.

I: “Why not?”

R: “Because the sense of solidarity among the Ghanaians is different than among the Antilleans. We’re also very close, but just with family. [With the Ghanaians,] they’re all brothers and sisters. [With us,] your immediate family is what counts, and the rest are just acquaintances”. (Antillean key figure)

Excerpts from the Antillean and Surinamese mothers echoed the explanations of the key figures. They more frequently mentioned the individual-oriented channels through which they are normally reached, most of which were not ethnically specific. For instance, Surinamese mothers mentioned social media and email. Other alternative channels they used were health care, brochures by mail, word-of-mouth communication with friends and colleagues, or possibly via Surinamese radio and television. However, the mothers who were reached via “an Antillean radio broadcast” and “Surinamese women’s organizations” were in fact reached by an immediate family member who was the leader/radio producer, or by a friend.

The interviewees, both mothers and key figures, linked the role of the available organizations to the ethnic communities’ characteristics and gave this as the final explanation for reach. Excerpts from the Ghanaian interviews showed that they see ethnically specific community organizations as a “bridge to Dutch society”. The Ghanaian gatherings provided support, help, and advice about how to deal with specifically Dutch situations. Leaders of Ghanaian women’s organizations were described as being in touch with Dutch organizations such as the Public Health Service and the local government district. They invited experts to provide information to their group, and translated that information. The organizations were the usual channels for their members to get information about important issues, such as health. The Ghanaian key figures said these are what explained high attendance at their meetings.

I: “How do you learn more about activities that take place in the neighborhood?”

R: “Umm, [our Ghanaian women’s organization] has a secretary, and she looks all over to see where we can go”.

I: “But is there also another way for you to get information?”

R: “No, she gives us all of the information, and sometimes she just puts it on the table. Should we do this? Do we want to do this?”

I: “But don’t you get a newsletter from your local government district, for example?”

R: “Sure, a newsletter, all kinds of things. [The key figure] also reads them to us and explains it to those who don’t understand Dutch. (…) She [also] usually works together with the district”. (Ghanaian mother)

I: “What struck me was that quite a few young Ghanaian women get together at [names of women’s organizations] and in churches. Why do you think this is?”

R: “The reason for this is [for example], for most women (…), the communication between them and their partner isn’t like in Ghana. One of them works in the evening and the other one in the afternoon, so then you don’t see each other. So with a group like this they have more of a chance to talk about problems and give each other advice”. (Ghanaian key figure)

The one Antillean church played the same role, providing information every week about upcoming activities. In contrast, at ad hoc informational meetings organized by Surinamese organizations, no mothers with young children were present. Furthermore, a key figure mentioned that daughters of their elderly members said they were “*glad their mothers have Surinamese organizations to empower them,*” but they did not seem to be interested in these organizations themselves. Surinamese mothers said they were not used to receiving information about activities like this exercise program through their churches.

### Receptivity and participation: contribution of ethnically matched key figure versus ethnic Dutch health educator recruiters

If we compare expressions of receptivity and participation numbers between mothers exposed to ethnically matched recruiters with those exposed to ethnic Dutch health educators, overall, the former were better received and a larger proportion enrolled in the exercise classes. In the intervention condition, a more positive atmosphere was observed than in the comparison condition. Mothers seemed more open to and to pay more attention to the recruiter and recruitment message. The interviews with mothers supported this: they remembered the recruiter and which information she had given better if this was done by a key figure than by a health educator. Observed interaction was more positive, and the women asked more questions. Moreover, if Dutch health educators supported the information giving while key figures were involved in the recruitment, the health educator received more positive reactions, such as “*A really good initiative*”, “*It was a very nice evening”,* and *“Can you please come to our church to tell about this?”* In the comparison condition, by contrast, women left early or the health educator herself had to approach them.

We saw that almost all mothers who indicated they did not want to join the exercise program (or who indicated they might want to join but were not reached afterwards) had been recruited by a health educator (comparison condition; 9 of 10 mothers who refused to participate) (Table 4). There were a few more mothers in the intervention condition (12 of 35) than in the comparison condition (7 of 24) who indicated they wanted to join the exercise classes but ultimately did not. However, most mothers in the intervention condition (22 of 35) versus those in the comparison condition (7 of 24) actually started taking part in the exercise classes.

**Table 4:** Participation according to intervention versus comparison condition regarding recruiter

	**Total reach**	**Maybe → no**	**Maybe → not reached**	**Yes → no enrollment**	**Yes → participant**
**Total: intervention group - ethnically matched recruiter (I)**	32 + 3 word of mouth/enrolled later	-	1 (3%)	12 (34%)	19 + 3 (63%)
**Total: comparison group - Dutch health educator (C)**	22 + 2 word of mouth	4 + 1 (21%)	4 (17%)	7 (29%)	7 + 1 (33%)
					
**Pair 1:**					
(I) Ghanaian church 1	5 + 1 via another organization	-	-	3	2 + 1
(C) Ghanaian church 2	7	-	2	2	3
**Pair 2:**					
(I) Ghanaian women’s organization 1	19 + 1 enrolled later	-	-	5	14 + 1
(C) Ghanaian women’s organization 2	5	-	-	4	1
**Pair 3:**					
(I) Surinamese women’s organization 1	2	-	-	2	-
(C) Surinamese women’s organization 2	2	2	-	-	-
**Pair 4:**					
(I) Surinamese women’s organization 3	-	-	-	-	-
(C) Surinamese women’s organization 4	+1 via mother	+1	-	-	-
**Pair 5:**					
(I) Surinamese radio 1	-	-	-	-	-
(C) Surinamese radio 2	-	-	-	-	-
**Pair 6:**					
(I) Antillean radio (interview)	1 + 1 Surinamese via colleague	-	-	-	1 + 1
(C) Caribbean radio	1	-	1	-	-
**Pair 7:**					
(I) Antillean church	5	-	1	2	2
(C) Community project	5 + 1 via colleague	1	-	1	3 + 1
(C) Immigrant organization via a school	2	1	1	-	-

Excerpts of the interviewees described why they thought the ethnically matched recruiter achieved higher receptivity and participation in the intervention condition. First, the recruiter was similar and familiar to the women, which seemed to create openness, attention (e.g., better recall of the recruiter and her message), accessibility (e.g., actively approach the key figure for more information), trust, and persuasion, regardless of ethnic group. Explanations given for this had to do with the recruiter’s ethnicity and whether she was known for her expertise or activities in this area. If the recruiter was described as a close, familiar person, this was said to foster trust and persuasion.

R: “I thought the radio spot was good, too. Because everyone called me, saying, ‘[name of key figure], are you going to take part in a project?’ Because they heard my name (…)”.

I: “Ooh, okay, that’s funny, because I actually didn’t have a single response to what was on the radio. But you did, huh?”

R: “Yes, I did, I heard it, yes. But then, you’re not so well known in the Surinamese community. So I’m not too surprised. But the moment you mentioned my name. Then they say, ‘Hey [name of key figure], I heard your name on the radio. Are you taking part?’ I say, ‘Yes I am, together with Ms. [name of the health educator], we’re doing a project called Big Move *mama*.’” (Surinamese key figure, intervention condition)

I: “And who was the person who told you about Big Move *mama*? Who was that?”

R: “Er, er, [name of key figure], yeah, and also a lady, a white lady, but I forgot her name. Because [name of key figure] is… (runs her hand over her skin), so then I won’t forget, ha-ha-ha. So the other lady, she’s also white, but I forgot her name”.

I: “Well, it was also a while back. And [name of key figure], is she from the church?”

R: “Yes! The church, we have two services, one in the morning and one in the afternoon. But she goes to the morning service and I go to the afternoon service. But sometimes she comes to give us information”. (Ghanaian mother, intervention condition)

I: “And so is it also important that it’s your father [an Antillean radio producer] who’s saying this? Do you immediately take it on board then?”

R: “No (laughs), no, I don’t, but still…I have a really good relationship with my father, so as far as that goes. If he suggests something he thinks would be good for me, I’ll take it on board. That’s what I mean”. (Antillean mother, intervention condition)

Second, ethnically matched recruiters used the mother tongue to translate and explain things in a way that was relevant; this might have increased attractiveness, attention, and accessibility, although it did not seem just a matter of understanding. In fact, mothers and key figures mentioned that most Ghanaian and Antillean women could understand both English and Dutch, the languages used by the health educators. It may be, rather, the attraction to something “familiar”, as described by a key figure, and having no barrier to ask questions and interact. More positive interaction was observed when an ethnically matched recruiter used the mother tongue during recruitment, and if women approached these key figures afterwards with additional questions and for advice, they used their own language then as well. Although all women said their leader was able to translate if the Dutch health educators were the recruiters, questions were asked only in their native language if the information had also been given in this language.

I: “And I also noticed you were quick to translate it into Papiamento”.

R: “I translated it myself – it carries more feeling, the Antillean feeling. It has nothing to do with people not being able to understand Dutch or anything like that. It’s all about feeling – you feel more”. (Antillean key figure, intervention condition)

Third, mothers and key figures described, particularly, the ethnically matched recruiter as a “motivator”. The key figure was most frequently a role model, expressing a positive attitude towards exercising and the exercise program, modeling positive interaction with the health educator, and encouraging participation in the program (“*Let’s go with the group*”, “*Take this opportunity*”). Ghanaian women responded mainly with a wait-and-see attitude to the health educators’ interactive approach. When the key figure modeled positive interaction with the health educators, the whole group responded with questions and more positive reactions to the health educators afterwards. Subsequently, the availability of a role model who participated in the exercise classes herself and who could be a contact person if constraints emerged during enrollment seemed to contribute to actual participation after indicating interest. This was observed and also explained by a Ghanaian key figure:

R: “Yes, I think the information provided is good, I introduced you to the parents, you told the parents the reason, everything, and, er, you also did a good job, you talked about everything, so, er, people [responded] very enthusiastically and just signed up, and I wanted to take part too, so we had a good response (…)”.

I: “What was it that made everyone want to take part?”

R: “Because I told them it’s for our health and that it would be once a week and that we’d do it with the group. Like that, which was good. [But then later on] ‘the time is changed. So now you come from half past seven to half past eight’, I think it’s a little on the late side, but in fact I said no, I can’t complain. Because if I complain then the women will complain, too. Because everyone looks over at me (laughs)!” (Ghanaian key figure, intervention condition)

Another motivational approach was adaptation of the message by the matched recruiter, unconsciously towards cultural factors and consciously towards mothers’ characteristics and the social network. For example, an Antillean recruiter emphasized that she exercises so she can eat and also stressed the social benefits of exercising with others. Moreover, there were Antillean and Surinamese recruiters who tailored the benefits message to the mothers they knew personally and included such benefits as less back pain, exercising, and some time for oneself. Further, they were able to link people within social networks to each other to exercise together. This seemed to result in greater participation by the mothers reached, especially those mothers who at first were skeptical about the benefits of exercising.

Nevertheless, providing information was not always the best role for an ethnically matched recruiter. There were several examples where incomplete or even inaccurate information was given, which did not benefit participation and was counterproductive for receptivity. For example, in a comparison condition church, the information meeting was inaccurately presented as having a health and a healthy diet focus instead of the health benefits of exercising and an invitation for Big Move *mama*. Although the people listened they were not open to the information and had negative attitudes and reactions afterwards (“*This wasn’t what we expected”*).

Because of this, key figures generally preferred to have a health educator give the most important information about exercising and Big Move *mama*; the mothers also appreciated this. Only if the recruiter was a health expert herself or was known for her sport activities she felt to be capable of informing and recruiting mothers. Therefore, several key figures from the intervention condition asked the health educator to provide the information, since she was the expert. The Public Health Service of Amsterdam was perceived as a credible source – also because of collaborations with community organizations in the past – thereby creating trust in the exercise program offered. Mothers perceived the health educator as a suitable messenger, especially because she had the knowledge required and she was able to answer questions in detail. Further, the health educator was trusted because of her expertise and her involvement with the target group, her friendliness, and her patience in explaining the program. By hearing from a trusted expert, the women could assess the program for themselves and determine their own feelings about it.

R: “I think it’s good that someone from the Public Health Service does it. (…) See, to me, I just see someone from the GGD, you know, they’re not specialized in one thing, but they know a little bit of everything. [Or] she can also give you information along the lines of, ‘my colleague can help you further, ’ that kind of thing. So I do like it that it’s someone from the GGD”.

I: “And what about someone from the neighborhood?”

R: “Yes, that’s a possibility, but then I hope that person doesn’t get stuck if someone has questions they can’t answer. That comes over as rather unprofessional. So… no, I was glad she was also from the GGD”. (Surinamese mother, comparison condition)

I: “And why was it important to you that I explained things?”

R: “Well, because (…) you know more about the subject matter than I do. And the only thing I can help with is introducing you to the women. Emmm, I say a little and then, er, you can tell them about it yourself, and I always think that works the best with us, then they know that the person or the expert is getting the message across or giving the information, and then they can [ask] all of their questions (…) And the women felt much the same, they accepted it, so that was good”. (Ghanaian key figure, intervention condition)

## Discussion

Program recruitment through ethnically specific community channels was successful among Ghanaian mothers but less so among Antillean and Surinamese mothers. The more close-knit an ethnic community was, retaining their own culture and with poorer comprehension of the Dutch language, the better mothers were reached through ethnically specific organizations. They came together more often in organizations that had regular meetings with fixed groups and included young women; these organizations were a bridge to Dutch society and services, such as to the Public Health Service of Amsterdam and its health promotion facilities. Furthermore, regardless of ethnic group, we found that using ethnically matched recruiters resulted in higher receptivity, and, among the Ghanaian mothers in particular, greater participation. This was explained by the fact that the ethnically matched recruiter was a familiar, trusted person, a translator, and a motivator because of her enthusiasm and encouragement or message adaptation. To increase recruitment credibility and professionalism, key figures and mothers preferred the ethnic Dutch health expert.

The success of using ethnically specific organizations to reach mothers seems to depend on the target community’s characteristics. Our findings reinforce previous findings in that, in the Netherlands, Ghanaians, a more close-knit community, seem to rely mainly on their own channels, while the Surinamese seemed to do this the least (Hartman, Nierkens, Cremer, Verhoeff, Stronks: Is channel segmentation necessary to reach a multiethnic population with weight-related health promotion? An analysis of use and perception of communication channels, submitted). This is supported by a study in England that reported differences in responses to recruitment efforts via the ethnic community that were related to the social network structure, such as how close-knit it was [28]. This characteristic might, therefore, apply in different contexts and different ethnic groups. Our study adds further insight into conditions under which use of ethnically specific channels, seemingly related to ethnic community characteristics and associated organization characteristics, could be successful.

In addition, this study provided insight into which of the roles of the ethnically matched key figures might enhance recruitment and seemed to relate to the confidence of the individual in the role. These roles included familiar and trusted “natural helpers”, translators, and positive role models. These findings support previous results on the effectiveness of community health workers and effectiveness of their ability to provide tailored and culturally relevant messages [10,29]. We found that organizational leaders who were employed as recruiters were less likely to do the information giving, however. Only a few key figures felt capable of being fully responsible for recruitment. Although community health workers might contribute to recruitment by being seen as a trustworthy person, an expert was also needed to provide the information [9].

The strength of a process evaluation like this one is that it reveals underlying mechanisms through which interventions influence outcomes, for whom, why, and under which conditions [30]. This small-scale process evaluation was conducted without predefined hypotheses in a real-life setting dealing with community practice. This limited the researchers’ control of the number of mothers reached, what influenced the evaluation of research question 2, the degree to which perfect matching of community organizations was possible, and the degree to which key figures accepted the role of community health worker recruiting mothers for the exercise program. However, that the methods used could be adapted to real-life situations throughout the study is an advantage of the small-scale design. For instance, in cases where the organizational leader required support from a health educator, we could determine, by condition, how the collaboration affected receptivity and participation.

We recognize that this study design does not allow us to test for statistical significance the quantitative data collected on reach and participation methods. It was our intention that the quantitative data only provide a first impression of whether these methods might contribute to reach. This impression, then, was further explained by the qualitative data, which could contradict, confirm or complement. This process of using various types of data, also known as triangulation, contributes to a better understanding of a given phenomenon (i.e., internal validity) [31].

Finally, as a consequence of the higher reach among Ghanaian mothers, we have more evidence that those key figures can contribute to higher receptivity and participation than among Surinamese and Antillean mothers. In all three ethnic groups, however, qualitative data indicated increased receptivity when key figures were involved. We cannot draw firm conclusions on whether the key figures needed to be ethnically matched. Nevertheless, some explanations for receptivity related to ethnicity-related characteristics of the key figure, skin color and language, according to Ghanaians and Antilleans. We found the strongest indications among the Ghanaian group though, since for them the ethnically specific community organizations were their main channels, and the leaders had prominent roles.

Further research on ethnic minority groups that are not as close-knit, that are less organized, and that have local language proficiency is warranted to explore reach and to determine key figure characteristics that promote receptivity and participation. Furthermore, a next step would be to conduct a larger scale effectiveness study to test the actual contribution of ethnicity-based targeted recruitment approaches among close-knit ethnic communities that retain their own culture and language. In these studies the use of general audience–oriented channels would be needed along with the ethnically specific channels as a comparison group.

## Conclusions

The use of ethnically specific organizations resulted in a successful recruitment for an exercise program in close-knit, highly organized ethnic communities with limited fluency in the local language. Ethnically matched recruiters were most confident being motivators, translators, and positive role models. However, they often needed an expert (health educator) for effective information delivery. In particular the involvement of a trusted familiar key figure in combination with the expert during recruitment might have contributed to the increased receptivity and participation in the exercise program.

## Competing interests

The authors declare that they have no competing interests.

## Authors’ contributions

MAH collected the data, performed the data extraction, analyzed the data, and drafted the manuscript. VN, SWC, KS, and APV participated in interpreting the results, and critically revised the intellectual content of the manuscript. All authors read and approved the final manuscript.

## Acknowledgements

We would like to thank Renée Corstjens and Gwen van Husen, health promoters of the Public Health Service, for their help in the preparation phase of this recruitment study. Thanks to Linda Boateng for her intermediary role with the Ghanaian churches, and to health educators Tamara Vincent and Chantal Goor for their help with several of the comparison groups. We are grateful to the leaders of the community organizations for their collaboration during the recruitment and input for the evaluation. Thanks to Lotte de Schouwer, Arja Schreij, and Maria Besteman for interviewing the mothers and to these mothers for participating. We would like to acknowledge Charlotte Vissenberg for her role as second coder. Finally, we acknowledge contributors of the Health Promotion and Behavioral Sciences Doctoral/Post-Doctoral Research Seminar (UTHealth) for their input in the final revisions of the manuscript.

## Pre-publication history

The pre-publication history for this paper can be accessed here:

http://www.biomedcentral.com/1471-2458/13/768/prepub
